# Integration of the Renal Angina Index and Urine Neutrophil Gelatinase-Associated Lipocalin Improves Severe Acute Kidney Injury Prediction in Critically Ill Children and Young Adults

**DOI:** 10.1016/j.ekir.2022.05.021

**Published:** 2022-05-25

**Authors:** Stuart L. Goldstein, Kelli A. Krallman, Cassie Kirby, Jean-Philippe Roy, Michaela Collins, Kaylee Fox, Alexandra Schmerge, Sarah Wilder, Bradley Gerhardt, Ranjit Chima, Rajit K. Basu, Lakhmir Chawla, Lin Fei

**Affiliations:** 1 Center for Acute Care Nephrology, Cincinnati Children’s Hospital, Cincinnati, Ohio, USA; 2 Department of Pediatrics, Division of Nephrology and Hypertension, Cincinnati Children’s Hospital Medical Center, Cincinnati, Ohio, USA; 3 Sainte Justine Hospital, Montreal, Canada; 4 Department of Pediatrics, Division of Critical Care Medicine, Cincinnati Children’s Hospital Medical Center, Cincinnati, Ohio, USA; 5 Ann and Robert H. Lurie Children’s Hospital of Chicago, Chicago, Illinois, USA; 6 Intensive Care Medicine, Veterans Health Administration, Washington DC, USA; 7 Division of Biostatistics and Epidemiology, Cincinnati Children’s Hospital Medical Center, Cincinnati, Ohio, USA; 8 Department of Pediatrics, University of Cincinnati College of Medicine, Cincinnati, Ohio, USA

**Keywords:** acute kidney injury, neutrophil gelatinase-associated lipocalin, renal angina index, severe AKI

## Abstract

**Introduction:**

Acute kidney injury (AKI) occurs in one-fourth of children and young adults admitted to pediatric intensive care unit (PICU). Severe AKI (sAKI; Kidney Disease: Improving Global Outcomes stage 2 or 3) is associated with morbidity and mortality. An AKI risk stratification system, the Renal Angina Index (RAI) calculated at 12 hours of admission, exhibits excellent performance to rule out sAKI at 72 hours of admission. We found that integration of urine neutrophil gelatinase-associated lipocalin (NGAL) with RAI improves prediction of sAKI. We now report the first-year results after implementation of our prospective automated RAI-NGAL clinical decision support (CDS) program.

**Methods:**

Patients 3 months to 25 years of age were eligible. Admission order sets have a conditional order for urine NGAL released when a 12-hour RAI ≥8. The primary outcome was sAKI any time at days 2 to 4 of admission. We assessed performance of the RAI and RAI+/NGAL to predict the primary outcome.

**Results:**

A total of 1427 unique patients accounted for 1575 admissions. In 147 admissions, RAI was ≥8. RAI <8 had negative predictive value (NPV) of 0.98 (95% CI 0.97–0.99); RAI ≥ 8 had positive predictive value (PPV) of 0.37 (95% CI 0.30–0.46) to predict days 2 to 4 sAKI (area under the receiver operating characteristic curve [AUC-ROC] 0.88 [95% CI 0.84–0.92]). Of 147 RAI+ patients, 89 had NGAL available. RAI/NGAL combination improved PPV (0.64, 95% CI 0.50–0.79) without decrement in NPV (0.98, 95% CI 0.97–0.98).

**Conclusion:**

AKI biomarker assessment directed by risk stratification improves prediction of sAKI in critically ill children and young adults. This CDS process has potential to enrich the population for interventional study, although improvement to adherence to CDS is needed.

## Graphical abstract

**Figure:**
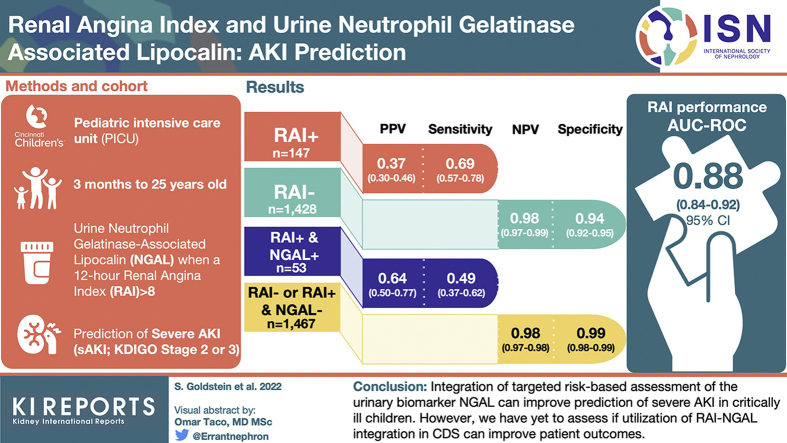

AKI is a common clinical problem with devastating consequences in critically ill children. AKI occurs in 10% to 40% of children admitted to a PICU, and sAKI (stage 2 or 3 as defined by the Kidney Disease: Improving Global Outcomes criteria)^1^ is independently associated with morbidity and mortality in critically ill children.^2^, ^3^, ^4^, ^5^, ^6^ Currently, only supportive measures such as kidney replacement therapy (KRT) can be offered to critically ill patients with AKI, and there is much debate as to when to initiate KRT, as clinicians have not had tools to accurately predict which patients will or will not develop sAKI. Lack of reliable AKI risk assessment represents a major gap in our ability to provide an invasive procedure in a timely manner for only those patients who need the therapy**.**

We have spent the past 10 years validating a pediatric AKI risk stratification system, the RAI.^7^ The RAI, when calculated 12 hours after PICU admission, has been evaluated to predict sAKI at 72 hours of PICU admission in single- and multicenter studies.^8^, ^9^, ^10^ A recent systematic review of 11 pediatric studies revealed an AUC-ROC of 0.88 (95% CI 0.85–0.91), sensitivity 0.85 (95% CI 0.74–0.92), and specificity 0.79 (95% CI 0.69–0.89).^11^ In a previous pilot study, we found that integration of the novel AKI biomarker NGAL with the RAI exhibited an AUC-ROC of 0.97 to predict sAKI in critically ill children at 72 hours.^12^

Here, we report the first-year results (July 1, 2018, to June 30, 2019) of our integrated RAI-NGAL CDS to predict sAKI presence at 72 hours of PICU admission. We hypothesized that integration of NGAL into the RAI would improve the PPV and specificity of sAKI prediction than the RAI alone. In addition, we report our CDS program performance with respect to implementation in this time frame.

## Methods

The methods for this prospective Trial in AKI using NGAL and Fluid Overload to optimize CRRT Use (TAKING FOCUS 2) have been reported elsewhere.^13^ Briefly, all patients 3 months to 25 years of age admitted to the Cincinnati Children’s Hospital Medical Center PICU were eligible for enrolment. We used an upper age limit of 25 years as children with chronic illness are often cared for until age 25 years when they mature to young adult age. Exclusions included subjects admitted to PICU for <48 hours, history of baseline chronic kidney disease stage 4 or 5, an active do not resuscitate order or the clinical team is not committed to escalating medical care, or AKI requiring KRT before the PICU admission. The TAKING FOCUS 2 protocol was approved by the Cincinnati Children’s Hospital Medical Center Institutional Review Board, and as this was an observational study without an intervention, the study received a waiver of the need for informed consent. TAKING FOCUS 2 was registered on ClinicalTrials.gov (NCT03541785) before patient enrolment and is supported by the Cincinnati Children’s Hospital Innovation Fund and by a grant from the National Institute of Diabetes and Digestive and Kidney Diseases (P50 DK 096418-06). We used a REDCap database, a project supported by the National Center for Advancing Translational Sciences of the National Institutes of Health, under award number UL1TR000077. The content is solely the responsibility of the authors and does not necessarily represent the official views of the National Institutes of Health.

### Study Flow

**Figure 1:**
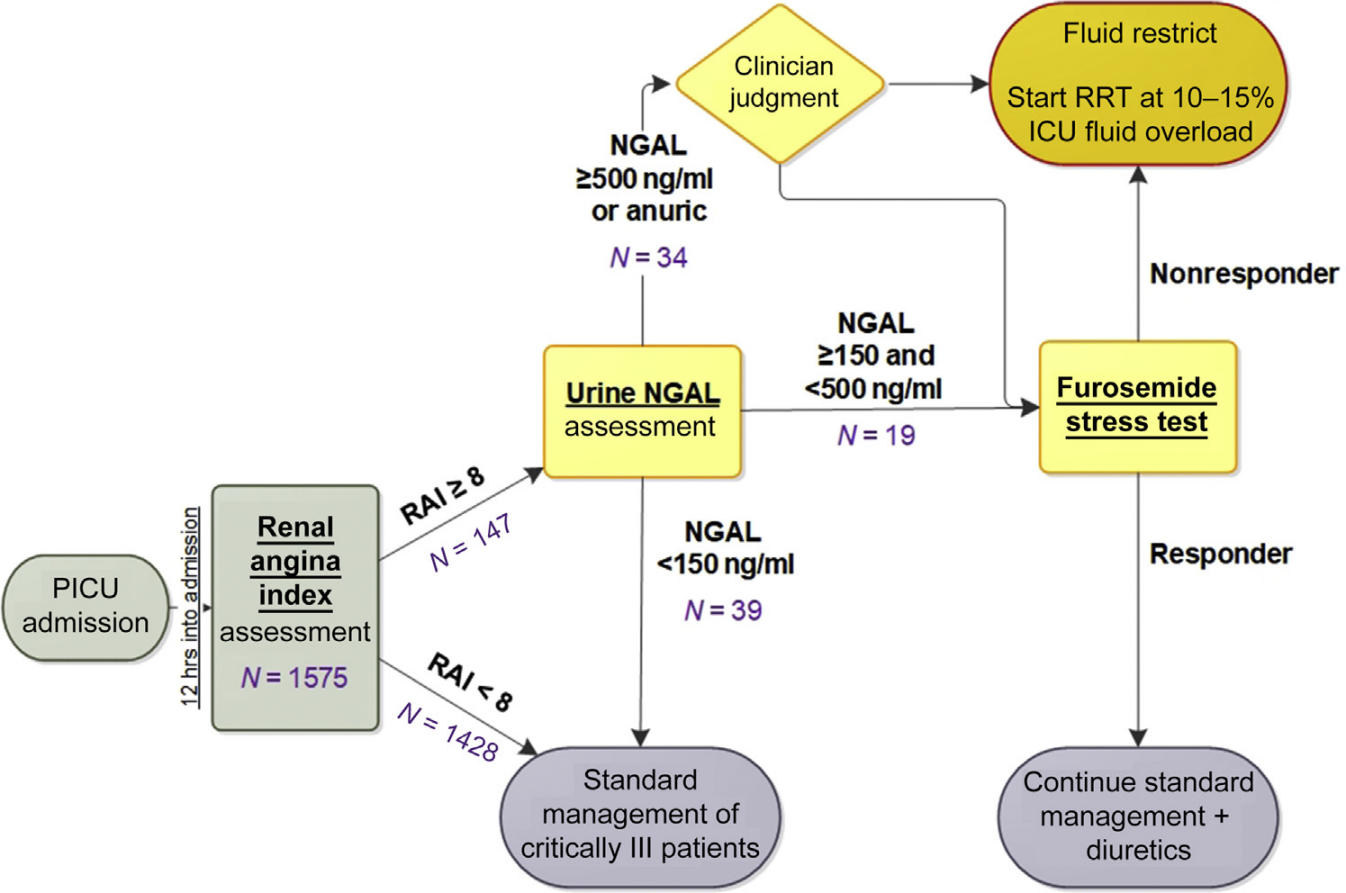
The TAKING FOCUS 2 AKI clinical decision support flow algorithm. The clinical support algorithm suggests that patients at low risk, RAI− and RAI+/NGAL−, receive standard management per PICU. Patient at high risk, RAI+/NGAL+, with NGAL 150−500 ng/ml, can have their risk further stratify with a FST, unless contraindicated, whereas those with >500 ng/ml can either have an FST or initiate RRT if there is an emergent indication or if it is deemed better/urgent by the primary team. FST responders have a lower risk of requiring RRT, as such, management with diuretic and fluid restriction is suggested, although FST nonresponders are likely to fail diuretic management and an initiation of RRT is suggested if FO >10% to 15% cannot be prevented by fluid restriction alone. The study flow is also illustrated in this figure. AKI, acute kidney injury; FO, fluid overload; FST, furosemide stress test; ICU, intensive care unit; NGAL, neutrophil gelatinase-associated lipocalin; PICU, pediatric intensive care unit; RAI, Renal Angina Index; RRT, renal replacement therapy.

The RAI-NGAL study flow is depicted in Figure 1. The RAI is automatically calculated at 12 hours after PICU admission and resulted in the electronic health record (Epic, Verona, WI). The RAI calculation has been published extensively. The RAI is the product of demographic risk (intensive care unit [ICU] admission = 1, stem cell or solid-organ transplant recipient = 3, invasive mechanical ventilation and 1 i.v. vasoactive medication = 5) and degrees of physiological change (increase in serum creatinine [SCr] or positive fluid accumulation [1, 2, 4, and 8]). The algorithm identifies a measured SCr value closest to 90 days before admission, within a ±1 month window, as the baseline SCr. If none is found, it imputes a baseline SCr based on the most recent patient height, within the last year, by assuming an estimated glomerular filtration rate of 120 ml/min per 1.73 m^2^, as validated in the pediatric literature.^6^^,^^14^ We used the bedside formula of Schwartz *et al.*^15^ for this imputation method:

Imputed SCr (mg/dl) = 0.413 × patient height (cm) / 120.

If no height is available, it uses patient age to impute a baseline SCr with a similar accuracy, as we have previously validated in the early stages of this project.^16^ If the RAI result is <8, the patient receives the routine clinical standard of care, and no further action is directed by the CDS pathway.

At Cincinnati Children’s Hospital Medical Center, all PICU admission orders have a conditional order for a urine NGAL placed that automatically releases if the RAI (range [1–40]) results at ≥8. At Cincinnati Children’s Hospital Medical Center’s clinical laboratory, urine NGAL is measured using the NGAL test assay (BioPorto Diagnostics, Denmark). Values were reported between <25 and 15,000 ng/ml by manual dilution. The NGAL result is available within 2 hours and automatically populated in the electronic health record.

Once the NGAL result is posted in the electronic health record, fluid, diuretic management, and initiation of KRT are guided by the CDS algorithm. If the NGAL result is <150 ng/ml, the patient receives the routine clinical standard of care, and no further action is directed by the CDS pathway. If the NGAL result is ≥500 ng/ml, then the PICU team will consider fluid restriction, a 1 mg/kg bolus infusion of furosemide (1.5 mg/kg if the patient has received a diuretic in the previous 24 hours), and/or initiation of KRT as needed to prevent fluid accumulation of >15% of body weight at PICU admission. If the NGAL result is 150 to 500 ng/ml, the patient will receive a 1 mg/kg bolus infusion of furosemide (1.5 mg/kg if the patient has received a diuretic in the previous 24 hours). If a patient is both RAI+ and NGAL+ (≥150 ng/ml), the research team communicates with the rounding ICU attending to remind them that the patient may be appropriate for the standardized furosemide assessment (furosemide stress test [FST]). Because patients need to be fluid replete before the FST, the RAI-NGAL CDS does not mandate it as the research team is not at the bedside. On the basis of the urine output response, the patient will either continue receiving diuretics or initiate KRT to prevent fluid accumulation of >15% of body weight at PICU admission. We chose these NGAL thresholds for the following reasons: (i) 25 ng/ml is the lower limit of detection for the BioPorto assay; (ii) 150 ng/ml has been associated with prediction of AKI in numerous pediatric populations; and (iii) 500 ng/ml has been associated with a high specificity for sAKI.^17^, ^18^, ^19^, ^20^

### Statistical Analysis

The data reported in this manuscript represent the first 2 stages of the TAKING FOCUS 2 CDS pathway: automated RAI calculation and RAI+-directed automatic NGAL ordering in the first year of the project. The primary outcome of interest is presence of stage 2 or 3 AKI at any time on days 2 to 4 of PICU admission. AKI was defined and staged by Kidney Disease: Improving Global Outcomes SCr (but not urine output) criteria or provision of KRT (stage 3),^1^ and the higher of either criterion was used for the outcome. We excluded patients admitted to our PICU who were older than 25 years of age (*n* = 38 [2.6%]) for the current analysis. Descriptive statistics are summarized for categorical and continuous variables. In assessing performance of predictive tests, that is, sensitivity, specificity, PPV, and NPV, we used exact CIs for binomial proportions. A NGAL concentration was presumed to be >500 ng/ml for patients who were anuric at 12 hours of ICU admission. The AUC-ROC was used to assess RAI predictive performance for sAKI (days 2–4). Fisher’s exact test is used to assess significance of binomial outcomes. Multiple logistic regression models incorporating both RAI or NGAL and Pediatric Risk of Mortality III score is used to predict sAKI (days 2–4). To account for serial correlation (multiple admissions) within patients, we used a generalized linear mixed effect model in the above-mentioned logistic regression where each patient specifies a random effect. We performed the following 2 sensitivity analyses: (i) using the first PICU admission for each unique patient (i.e., excluding any subsequent PICU admissions) and (ii) for the subset of patients who had measured (vs*.* imputed) baseline SCr concentration values.

For a small subset of patients (127 cases total) who had both RAI and NGAL measured, we performed net reclassification improvement and integrated discrimination improvement analyses to compare the performance improvement brought by NGAL over RAI positive alone. We do not report the outcomes of the FST in this current manuscript. All statistical significance is declared at the 0.05 level. The SAS software (9.4, Cary, NC) and R-packages pROC and Hmisc are used for statistical analyses.

## Results

The demographics and characteristics of the cohort is listed in Table 1, and the patient study flow is depicted in Figure 1. A total of 1427 unique patients (55.4% male) accounted for 1575 separate PICU admissions. Median patient age was 5.2 (interquartile range: 1.3–13.3) years. Mean patient estimated creatinine clearance at PICU admission was 149 ± 68 ml/min per 1.73 m^2^. Patient PICU mortality was 2.3% (95% CI: 1.6%–3.2%), and 28-day mortality was 2.9% (95% CI: 2.1%–4.0%).

**Table 1:** Demographics and characteristics of the TAKING FOCUS 2 cohort

Variables		
Individual patients (*n =* 1427)		
Sex	Female, *n* (%)	637 (44.6)
	Male, *n* (%)	790 (55.4)
Age (yr)		
	Mean (SD)	7.6 (6.9)
	Median (IQR)	5.2 (1.3–13.3)
	(Min, Max)	(0.16, 25)
Transplant—stem cell, *n* (%)		37 (2.6)
Transplant—solid organ, *n* (%)		55 (3.8)
Admissions (*n* = 1575)		
Primary admission diagnosis		
CNS, *n* (%)		180 (11.4)
Post-op/trauma, *n* (%)		474 (30.1)
Respiratory failure, *n* (%)		770 (48.9)
Pain, *n* (%)		140 (8.9)
Shock, *n* (%)		251 (15.9)
Cardiac, *n* (%)		37 (2.35)
Comorbidities		
GI, *n* (%)		327 (20.8)
Hematology-oncology, *n* (%)		215 (13.7)
Nephrology, *n* (%)		115 (7.3)
Pulmonary, *n* (%)		583 (37)
Kidney function at PICU admission		
Baseline serum creatinine (mg/dl)		
All 1575 admissions	Mean (SD)	0.34 (0.18)
	Median (IQR)	0.29 (0.22–0.46)
Admissions with measured value (*n* = 677)	Mean (SD)	0.31 (0.21)
	Median (IQR)	0.25 (0.17–0.39)
Admissions with imputed value (*n* = 898)	Mean (SD)	0.37 (0.13)
	Median (IQR)	0.34 (0.25–0.50)
Baseline eCCl (ml/min per 1.73 m^2^)		
All 1575 admissions	Mean (SD)	149 (68)
	Median (IQR)^a^	120 (120–155)
Admissions with measured SCr (*n* = 660)	Mean (SD)	187 (90)
	Median (IQR)	167 (127–230)
Admissions with imputed SCr (*n* = 882)	NA^a^	

CNS, central nervous system; eCCL, estimated creatinine clearance; GI, gastrointestinal; IQR, interquartile range; max, maximum; min, minimum; NA, not applicable; op, operation; PICU, pediatric intensive care unit; SCr, serum creatinine.

a NA because imputed eCCl is always 120 ml/min per 1.73 m^2^.

### Renal Angina Fulfillment and AKI

Of the 1575 PICU admissions, 80 (5.1%; 95% CI: 4.0%–6.3%) developed sAKI on days 2 to 4 of PICU admission. Of these admissions, 147 were RAI+ at 12 hours of PICU admission (9.3%; 95% CI: 7.9%–10.9%). Table 2 depicts the differences in underlying characteristics between patients who were RAI+ versus RAI−. Patients with a history of gastrointestinal, nephrological, or hematological/oncological comorbidities were more likely to be RAI+, whereas patients with an underlying respiratory comorbidity were less likely to be RAI+. Baseline SCr did not differ between RAI+ versus RAI− patients, but the estimated creatinine clearance was higher for RAI+ patients. Patients who were RAI+ were more likely to have days 2 to 4 sAKI than RAI− patients (55 of 147 [37.4%] vs. 25 of 1428 [1.8%], *P* < 0.0001). The rate of fluid accumulation to >10% did not differ between the groups.

**Table 2:** Demographic associations by RAI and NGAL status

Variables	RAI− (<8) (*n* = 1428), *n* (%)	RAI+ (≥8) (*n* = 147), *n* (%)	*P* value
CNS	176 (12)	10 (7)	0.06
Postsurgical or trauma	421 (29)	59 (39)	0.009
Respiratory failure	737 (50)	52 (35)	0.0003
Cardiac failure	27 (2)	10 (7)	0.001
Pain	128 (9)	15 (10)	0.65
Shock	201 (14)	56 (37)	<0.0001
Gastrointestinal	279 (19)	54 (36)	<0.0001
Hematology/oncology	185 (13)	45 (30)	<0.0001
Nephrological	105 (7)	21 (14)	0.006
Pulmonary	564 (39)	35 (23)	0.0002

	RAI− or RAI+ and NGAL− (<150 ng/ml) (*n* = 1467), *n* (%)	RAI+ (≥8) and NGAL+ (≥150 ng/ml) (*n* = 53), *n* (%)	
CNS	178 (12)	5 (9)	0.67
Postsurgical or Trauma	440 (29)	14 (26)	0.65
Respiratory failure	748 (50)	21 (38)	0.10
Cardiac failure	30 (2)	3 (6)	0.11
Pain	130 (9)	8 (14)	0.14
Shock	217 (14)	25 (37)	<0.0001
Gastrointestinal	293 (20)	22 (40)	0.0005
Hematology/oncology	193 (13)	22 (40)	<0.0001
Nephrological	109 (7)	15 (27)	<0.0001
Pulmonary	574 (38)	9 (16)	0.0009

CNS, central nervous system; NGAL, neutrophil gelatinase-associated lipocalin; RAI, Renal Angina Index.

**Figure 2:**
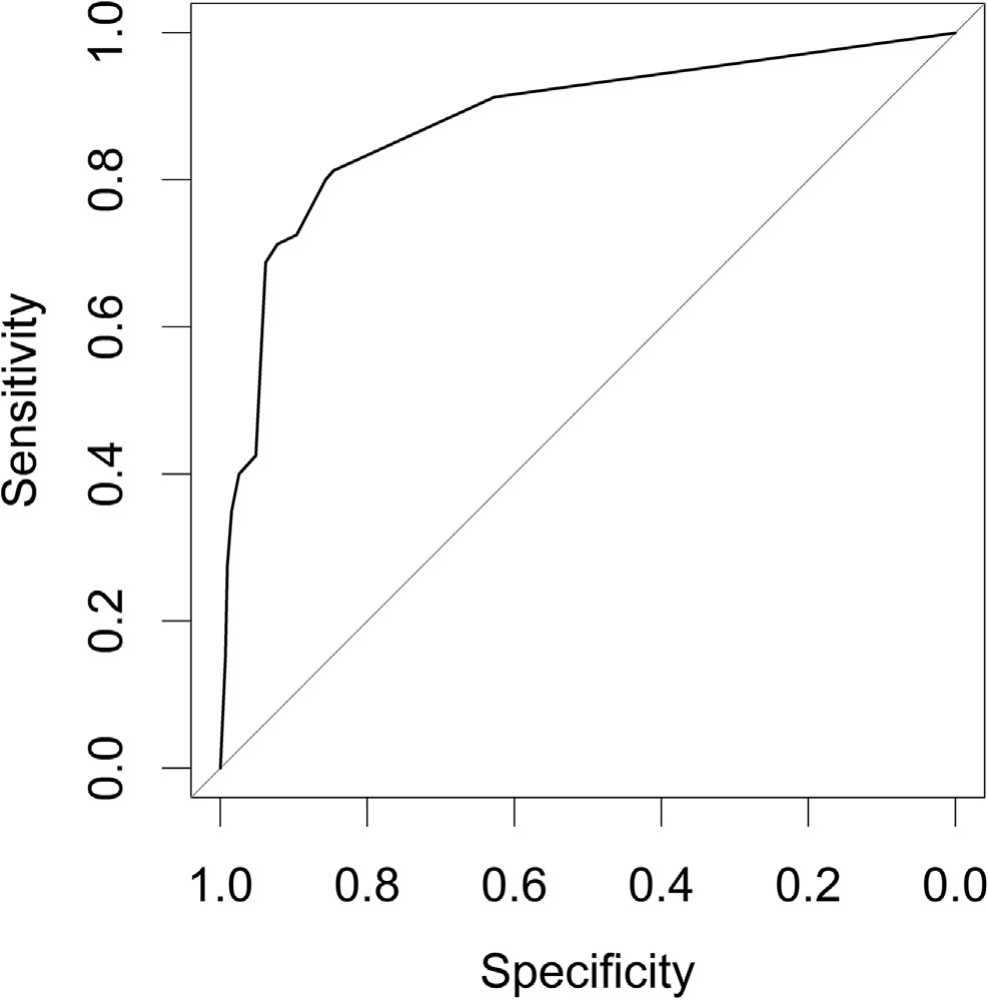
The AUC-ROC for the Renal Angina Index result at 12 hours of ICU admission to predict stage 2 or 3 AKI 2 to 4 days after ICU admission. AUC-ROC = 0.88 (95% CI 0.84–0.92). AKI, acute kidney injury; AUC-ROC, area under the receiver operating characteristic curve; ICU, intensive care unit.

The performance characteristics for the RAI to predict days 2 to 4 sAKI are displayed in Table 3. A RAI <8 had a NPV of 0.98 (95% CI: 0.97–0.99) with specificity of 0.94 (95% CI: 0.92–0.95), and an RAI ≥8 had a PPV of 0.37 (95% CI: 0.30–0.46) with sensitivity of 0.68 (95% CI: 0.57–0.78). The 12-hour RAI predicted days 2 to 4 sAKI with an AUC of 0.88 (95% CI: 0.84–0.92). (Table 3 and Figure 2).

**Table 3:** RAI and NGAL performance characteristics to predict days 2 to 4 sAKI

Tested result	*n*	Predicted values				RAI performance AUC-ROC (95% CI)
		PPV (sAKI+ D 2∼4)	Sensitivity (sAKI+ D 2∼4)	NPV (sAKI- D 2∼4)	Specificity (sAKI- D 2∼4)	
RAI+	147	0.37 (0.30–0.46)	0.69 (0.57–0.78)			0.88 (0.84–0.92)
RAI−	1428			0.98 (0.97–0.99)	0.94 (0.92–0.95)	
RAI+ and NGAL+	53	0.64 (0.50–0.77)	0.49 (0.37–0.62)			
RAI− or RAI+ and NGAL−	1467			0.98 (0.97–0.98)	0.99 (0.98–0.99)	

AKI, acute kidney injury; AUC-ROC, area under the receiver operating characteristic curve; NGAL, neutrophil gelatinase-associated lipocalin; NPV, negative predictive value; PPV, positive predictive value; RAI, Renal Angina Index; sAKI, severe AKI.

### Incorporation of NGAL

A urine NGAL result was available (or the patient was anuric, so NGAL was assumed to be >500 ng/ml, *n =* 6) for 92 of the 147 RAI+ patient admissions (62.6%). The most common reasons for unavailability of an NGAL result were as follows: RAI incorrectly calculated as <8 resulting in error (79.3%), NGAL order not included in admission order set (8.6%), and not enough urine produced to send for NGAL test (5.2%). The RAI calculation error occurred for patients with recent transplant that had not been entered into the patient diagnosis list or inability to calculate estimated creatinine clearance because of missing patient height (which was corrected early in the study course^16^). The distribution of NGAL concentrations was <150 ng/ml (*n =* 39), 150–499 mg/ml (*n =* 19), and >500 ng/ml (*n* = 34). Demographic data for RAI-positive patients stratified by the these 3 different NGAL ranges are shown in Supplementary Table S1. Additional 41 (2.9%) RAI− patients had an NGAL result as part of PICU attending physician discretion, but these were not included in any of the analysis (as they were not part of the CDS pathway) except for the small subset analysis at the end of patients who had both an RAI and NGAL available, irrespective of RAI status.

Patients without a urine NGAL available were younger (median age 11.7 [interquartile range: 1.5–14.8] vs*.*12.1 [0.26, 23.6] years, *P* = 0.053) and had a lower sAKI rate (27.9% vs. 44.2%, *P* = 0.057) than patients who did not have an NGAL result available. There was no significant difference in Pediatric Risk of Mortality III score, sex, PICU mortality, or 28-day mortality between ICU admission with and without an NGAL measurement.

Table 2 depicts the differences in underlying characteristics between patients who were RAI+/NGAL+ and patients who were either RAI− or RAI+/NGAL−. Patients who were RAI+/NGAL+ were more likely to have an admission diagnosis of shock and gastrointestinal, hematological-oncological-nephrological comorbidities, but not pulmonary comorbidities. Patients who were RAI+/NGAL+ had a higher baseline SCr (but not lower estimated creatinine clearance) and higher Pediatric Risk of Mortality III scores. RAI+/NGAL+ patients were more likely to have days 2 to 4 sAKI than those who were RAI− or RAI+/NGAL− (34/53 [64.2%] vs. 35/1467 [2.4%], *P* < 0.0001).

The performance characteristics for NGAL incorporation into the RAI to predict days 2 to 4 sAKI are displayed in Table 3. The RAI/NGAL combination displayed an improved PPV of 0.64 (95% CI: 0.50–0.77) without a decrement in NPV of 0.98 (95% CI: 0.97–0.99) compared with the RAI alone. This improvement is also statistically significant because the lower bound of the 95% CI of 0.50 is higher than RAI alone PPV of 0.37 (95% CI: 0.30–0.46). Furthermore, integration of NGAL values in the small subset (*n =* 127) with both RAI and NGAL results revealed improvement in sAKI prediction over RAI alone (net reclassification improvement for this comparison: 0.760 [0.422–1.105], *P* < 0.0001; integrated discrimination improvement for this comparison: 0.0826 [0.0338–0.1315], *P* = 0.0009).

The results of the sensitivity analyses for each unique patient first ICU admission (*n =* 1427) and only patients with a measured baseline SCr (*n =* 677) are illustrated in Supplementary Tables S2 and S3. We observed similar improvements specificity and PPV in these subcohorts as we did in the analysis of the entire cohort previously discussed.

### Secondary Outcomes

The assessment of secondary outcomes is depicted in Table 4. Patients who were RAI+/NGAL+ had longer PICU and hospital LOS and increased PICU and 28-day mortality rates than patients who were either RAI− or RAI+/NGAL−. No difference was observed between any of the group comparisons with respect to development of ≥10% fluid accumulation.

**Table 4:** Outcome associations by RAI and NGAL status

Variables		RAI− (*n* = 1428)	RAI+ (*n* = 147)	Overall (*N* = 1525)	*P* value
D 2–4 sAKI, *n* (%)		25 (1.8)	55 (37.4)	80 (5.1)	<0.0001
D 1–7 fluid accumulation,^a^*n* (%)		387 (31)	41 (27.9)	428 (27.2)	0.85
PICU LOS (d)	Median	3.7	5.6	3.7	<0.0001
	IQR	2.6–7.6	3.4–10.4	2.7–7.7	
Hospital LOS (d)	Median	8.5	16.6	8.7	<0.0001
	IQR	4.6–21.7	9.6–37.7	4.6–22.6	
PICU mortality	Alive, *n* (%)	1407 (98.5)	131 (89.1)	1538 (97.7)	<0.0001
	Deceased, *n* (%)	21 (1.5)	16 (10.9)	37 (2.3)	
28-d mortality	Alive, *n* (%)	1398 (97.9)	131 (89.1)	1529 (97.1)	<0.0001
	Deceased, *n* (%)	30 (2.1)	16 (10.9)	46 (2.9)	

Variables		RAI− or RAI+ and NGAL− (*n* = 1457)	RAI+ and NGAL+ (*n* = 53)	Overall (*N* = 1510)	*P* value
D 2–4 sAKI, *n* (%)		35 (2.4)	34 (64.2)	69 (4.5)	<0.0001
D 1−7 fluid accumulation,^a^*n* (%)		402 (27.4)	17 (32.1)	419 (27.6)	0.44
PICU LOS (d)	Median	3.7	6.6	3.7	0.0005
	IQR	2.6–7.59	3.53–13.6	2.61–7.65	
Hospital LOS (d)	Median	8.5	15.2	8.6	<0.0001
	IQR	4.6–21.7	10.4–36.1	4.6–22.4	
PICU mortality	Alive, *n* (%)	1443 (98.4)	44 (83.0)	1487 (97.8)	<0.0001
	Deceased, *n* (%)	24 (1.6)	9 (17.0)	33 (2.2)	
28-d mortality	Alive, *n* (%)	1434 (97.8)	45 (84.9)	1479 (97.3)	<0.0001
	Deceased, *n* (%)	33 (2.2)	8 (15.1)	41 (2.7)	

AKI, acute kidney injury; ICU, intensive care unit; IQR, interquartile range; LOS, length of stay; PICU, pediatric intensive care unit; NGAL, neutrophil gelatinase-associated lipocalin; RAI, Renal Angina Index; sAKI, severe AKI.

a Fluid overload is defined as >10% fluid accumulation based on ICU admission weight: %fluid accumulation = ([fluid volume In (l) – fluid volume Out (l)]/ICU admission weight (kg) × 100%.

## Discussion

This prospective single-center study builds on previous work to integrate a simple risk stratification system, the RAI, with a well-studied kidney tubular biomarker, NGAL, to improve sAKI prediction early during a child’s PICU admission. Our data reveal that addition of NGAL to the RAI increased the PPV and specificity for sAKI prediction, without a decrease in NPV.

The AUC-ROC of 0.88 for the RAI at 12 hours to predict days 2 to 4 sAKI is in line with previously published data and a recent systematic review of 11 studies.^11^ Importantly, the very good AUC for the RAI in this cohort is driven, as in all previous studies, by the NPV. Optimizing the NPV was the aim of our RAI construct, as we want to rule out AKI risk for most ICU patients, and direct clinicians to order a urinary AKI biomarker in only those patients who are truly at risk. The RAI+ rate of approximately 10% in the current study is comparable with previous pediatric studies, revealing the RAI can effectively rule out 90% of children for risk of sAKI early in the PICU course.

Undirected use of a biomarker outside of clinical context increases cost and will degrade its performance. For example, an elevated troponin is not specific for myocardial ischemia outside of the acute coronary syndrome context.^21^^,^^22^ Thus, providing clinicians with a readily available tool to direct biomarker assessment reliably will increase confidence in the value of the biomarker and potentially enhance its utility. Such confidence is crucial for translation to clinical care, because despite >2 decades of solid clinical research revealing the excellent performance of AKI biomarkers, manuscripts are still being published asking if it is “time for clinical implementation”^23^ or describing the state of the art as their “promise.”^24^

The current step for clinical application and acceptance of the RAI and NGAL was to integrate their use into the CDS. We reported the successful development and validation of a real-time RAI notification system at our center.^16^ Although the calculation of the RAI is simple, computation-based patient stratification systems such as the RAI must be reliable and reported in real time for clinician acceptance into the CDS. Traditional patient scoring systems rely on busy clinicians or research coordinators removed from the bedside for manual data extraction and tabulation. This manual process creates systematic vulnerabilities ranging from variable accuracy, time lag between patient presentation and screening, and increased costs to the patient and the medical/research system. Most importantly, these barriers lead to delays in patient stratification and identification.

The next step to maximize the value of the RAI-NGAL CDS model will be to direct interventions in RAI+/NGAL+ subjects to improve outcomes. Currently, the only care we can provide for patients with AKI is supportive, with fluid management strategies, avoidance of nephrotoxic medications when possible, and initiation of KRT. The second and third years of the TAKING FOCUS II project will evaluate CKRT initiation thresholds and use of the FST^25^ to further refine AKI risk assessment and direct care in children (Supplementary Figure S1). Integration of AKI risk and renal stress biomarkers to direct care bundles has been found to reduce AKI rates and severity in pilot studies of adults after surgery.^26^^,^^27^ We believe that similar improvements can be realized in critically ill children as well.

Although the RAI has consistently good to very good performance in critically ill children admitted to the PICU, we have always considered that it would need to be calibrated for specific populations.^7^ In fact, a recent modification of the RAI for critically ill adult patients observed similar performance to the base RAI when diabetes and sepsis were added to the model,^28^ and we improved RAI prediction for sAKI in children with sepsis/septic shock by adding platelet count into the model.^29^ We continue to entertain the likelihood that further calibration may be necessary to improve the performance to the RAI and its modification. However, because the RAI is calculated at 12 hours, and sepsis is often not in the problem list of diagnosis list at that time, it may be difficult to operationalize risk factors such as sepsis into a real-time CDS tool.

The current study has numerous strengths. It is a large prospective study of the RAI in all pediatric and young adult admissions to an ICU of >48 hours. The RAI was calculated automatically by programming of our electronic health record, and the NGAL order was ordered reflexively when that RAI resulted at ≥8. Thus, data extraction for analysis was easy, feasible, and reliable. We were able to validate 100% of the RAI and NGAL results before conducting the analysis.

There are limitations and challenges in our work that lead us to interpret our data with caution and pose barriers to potential generalizability. First, we did not assess NGAL concentrations in patients who were RAI negative. As such, we could not reliably calculate an AUC-ROC for the RAI-NGAL combination, that is, assessment of the overall change in predictive performance of adding NGAL to the RAI, irrespective of pretest probability. We had done this analysis in an earlier study, AKI-CHERUB, where such a design revealed AUC improvement from 0.80 to 0.97 (albeit in a small sample size).^12^ However, the TAKING FOCUS 2 strategy, as outlined previously, is to use biomarkers only in at-risk patients, as their performance should not be judged outside an intended use. Nevertheless, we recognize this limitation. Second, a strength of our approach also leads to some limitations. Reliance on the automatic calculation requires sustained reliable performance of the application. The first year of our work was devoted to ensuring the RAI application performance, which required multiple tweaks and continued manual surveillance by our research team to ensure accuracy. To that end, in the current report, a urine for NGAL was sent only in 61% of patients who were RAI+, with 85% of these misses resulting from incorrect automatic RAI calculation or lack of a conditional NGAL order in service-specific order sets. These errors have been rectified, and we expect to see increased rates of NGAL results in RAI+ patients. Third, the current RAI application is not readily transferable to other centers, and therefore, will require substantial investment in personnel time and cost for development and validation.

In conclusion, we have revealed that integration of targeted risk-based assessment of the urinary biomarker NGAL can improve prediction of sAKI in critically ill children. However, we have yet to assess whether utilization of RAI-NGAL integration in the CDS can improve patient outcomes. Achievement of this goal is the aim of our future work going forward.

## Supplementary Material

Supplementary File (PDF)

**Figure S1.** Timeline of the TAKING FOCUS II Project.

**Table S1.** Demographics for RAI+ patients stratified by urine NGAL concentration.

**Table S2.** RAI and NGAL performance characteristics for a first PICU admission.

**Table S3.** RAI and NGAL performance characteristics with a measured baseline serum creatinine.

## References

[1] Kidney Disease: Improving Global Outcomes (KDIGO) Acute Kidney Injury Work Group (2012). KDIGO clinical practice guideline for acute kidney injury. Kidney Int.

[2] Sutherland S.M., Byrnes J.J., Kothari M. (2015). AKI in hospitalized children: comparing the pRIFLE, AKIN, and KDIGO definitions. Clin J Am Soc Nephrol.

[3] Sanchez-Pinto L.N., Goldstein S.L., Schneider J.B., Khemani R.G. (2015). Association between progression and improvement of acute kidney injury and mortality in critically ill children. Pediatr Crit Care Med.

[4] Akcan-Arikan A., Zappitelli M., Loftis L.L. (2007). Modified RIFLE criteria in critically ill children with acute kidney injury. Kidney Int.

[5] Schneider J., Khemani R., Grushkin C., Bart R. (2010). Serum creatinine as stratified in the RIFLE score for acute kidney injury is associated with mortality and length of stay for children in the pediatric intensive care unit. Crit Care Med.

[6] Kaddourah A., Basu R.K., Bagshaw S.M. (2017). Epidemiology of acute kidney injury in critically ill children and young adults. N Engl J Med.

[7] Goldstein S.L., Chawla L.S. (2010). Renal angina. Clin J Am Soc Nephrol.

[8] Basu R.K., Zappitelli M., Brunner L. (2014). Derivation and validation of the renal angina index to improve the prediction of acute kidney injury in critically ill children. Kidney Int.

[9] Basu R.K., Wang Y., Wong H.R. (2014). Incorporation of biomarkers with the renal angina index for prediction of severe AKI in critically ill children. Clin J Am Soc Nephrol.

[10] Basu R.K., Kaddourah A., Goldstein S.L., AWARE Study Investigators (2017). Assessment of a renal angina index for prediction of severe acute kidney injury in critically ill children: a multicentre, multinational, prospective observational study. Lancet Child Adolesc Health.

[11] Abbasi A., Mehdipour Rabori P., Farajollahi R. (2020). Discriminatory precision of renal angina index in predicting acute kidney injury in children; a systematic review and meta-analysis. Arch Acad Emerg Med.

[12] Menon S., Goldstein S.L., Mottes T. (2016). Urinary biomarker incorporation into the renal angina index early in intensive care unit admission optimizes acute kidney injury prediction in critically ill children: a prospective cohort study. Nephrol Dial Transplant.

[13] Roy J.P., Krallman K.A., Basu R.J. (2020). Early sequential risk stratification assessment to optimize fluid dosing, CRRT initiation and discontinuation in critically ill children with acute kidney injury: Taking Focus 2 process article. J Clin Trials.

[14] Zappitelli M., Parikh C.R., Akcan-Arikan A. (2008). Ascertainment and epidemiology of acute kidney injury varies with definition interpretation. Clin J Am Soc Nephrol.

[15] Schwartz G.J., Munoz A., Schneider M.F. (2009). New equations to estimate GFR in children with CKD. J Am Soc Nephrol.

[16] Roy J.P., Johnson C., Towne B. (2019). Use of height-independent baseline creatinine imputation method with renal angina index. Pediatr Nephrol.

[17] Haase M., Bellomo R., Devarajan P. (2009). Accuracy of neutrophil gelatinase-associated lipocalin (NGAL) in diagnosis and prognosis in acute kidney injury: a systematic review and meta-analysis. Am J Kidney Dis.

[18] Krawczeski C.D., Goldstein S.L., Woo J.G. (2011). Temporal relationship and predictive value of urinary acute kidney injury biomarkers after pediatric cardiopulmonary bypass. J Am Coll Cardiol.

[19] Zappitelli M., Washburn K.K., Arikan A.A. (2007). Urine neutrophil gelatinase-associated lipocalin is an early marker of acute kidney injury in critically ill children: a prospective cohort study. Crit Care.

[20] Haase M., Devarajan P., Haase-Fielitz A. (2011). The outcome of neutrophil gelatinase-associated lipocalin-positive subclinical acute kidney injury: a multicenter pooled analysis of prospective studies. J Am Coll Cardiol.

[21] Ammann P., Fehr T., Minder E.I. (2001). Elevation of troponin I in sepsis and septic shock. Intensive Care Med.

[22] Agewall S., Giannitsis E., Jernberg T., Katus H. (2011). Troponin elevation in coronary vs. non-coronary disease. Eur Heart J.

[23] Albert C., Haase M., Albert A. (2021). Biomarker-guided risk assessment for acute kidney injury: time for clinical implementation?. Ann Lab Med.

[24] Devarajan P. (2020). The current state of the art in acute kidney injury. Front Pediatr.

[25] Penk J., Gist K.M., Wald E.L. (2019). Furosemide response predicts acute kidney injury in children after cardiac surgery. J Thorac Cardiovasc Surg.

[26] Meersch M., Schmidt C., Hoffmeier A. (2017). Prevention of cardiac surgery-associated AKI by implementing the KDIGO guidelines in high risk patients identified by biomarkers: the PrevAKI randomized controlled trial. Intensive Care Med.

[27] Gocze I., Jauch D., Gotz M. (2018). Biomarker-guided intervention to prevent acute kidney injury after major surgery: the prospective randomized BigpAK study. Ann Surg.

[28] Ortiz-Soriano V., Kabir S., Claure-Del Granado R. (2021). Assessment of a modified renal angina index for AKI prediction in critically ill adults. Nephrol Dial Transplant.

[29] Stanski N., Wong H.R., Basu R. (2021). Recalibration of the renal angina index for pediatric septic shock. Kidney Int Rep.

