# Supplementary material for: Integration of the Renal Angina Index and Urine Neutrophil Gelatinase-Associated Lipocalin Improves Severe Acute Kidney Injury Prediction in Critically Ill Children and Young Adults
Source: Kidney Int Rep. 2022 May 25;7(8):1842–9. doi: 10.1016/j.ekir.2022.05.021 (PMC9366367; doi:10.1016/j.ekir.2022.05.021)
Supplement: Supplementary File (PDF) [file mmc1.pdf]

Supplementary Material

Supplementary Table 1 – Demographics for RAI+ Patients and Admissions Stratified by Urine NGAL Concentration

**Unique Patients**		**NGAL Concentration (ng/mL)**			**p-value**
Variable		**<150 (n=38)**	**150-499 (n=19)**	**>500 (n=32)**	
Gender	Female	16 (42.1%)	9 (47.4%)	13 (40.6%)	0.92
	Male	22 (57.9%)	10 (52.6%)	19 (59.4%)	
Age (years)					
	Mean (SD)	10.7 (7.05)	12.4 (7.59)	11.4 (6.41)	0.68
	Median (IQR)	12.1 (3.09, 16.8)	13.6 (3.84, 20.3)	11.7 (6.91, 16.2)	
	(Min, Max)	(0.26, 23.3)	(0.43, 21.4)	(0.61, 23.6)	
Transplant – Stem Cell		2 (5.26%)	2 (10.5%)	7 (21.9%)	0.11
Transplant - Solid Organ		10 (26.3%)	6 (31.6%)	8 (25%)	0.68
**Total Admissions**		**NGAL Concentration (ng/mL)**			
Variable		**<150 (n=39)**	**150-499 (n=19)**	**>500 (n=34)**	
CNS		2 (5.13%)	2 (10.5%)	2 (5.88%)	0.51
Post-op/Trauma		19 (48.7%)	6 (31.6%)	8 (23.5%)	0.76
Respiratory Failure		11 (28.2%)	6 (31.6%)	14 (41.2%)	0.09
Pain		2 (5.13%)	4 (21.1%)	4 (11.8%)	0.13
Shock		24 (61.5%)	9 (47.4%)	19 (55.9%)	<0.0001
Cardiac		3 (7.69%)	1 (5.26%)	2 (5.88%)	0.10
**Co-Morbidities**					
GI		14 (35.9%)	10 (52.6%)	12 (35.3%)	0.0004
Hematology-Oncology		8 (20.5%)	6 (31.6%)	14 (41.2%)	<0.0001

Nephrology	4 (10.3%)	4 (21.1%)	9 (26.5%)	0.0001
Pulmonary	29 (74.4%)	17 (89.5%)	28 (82.4%)	0.0004

Supplemental Table 2 –RAI and NGAL Performance Characteristics to Predict Day 2-4 Severe AKI (sAKI) for the first PICU admission to each unique patient.

Tested result	n	Predicted Values (95% CI)				RAI Performance
		PPV (sAKI+ D2~4)	Sensitivity (sAKI+ D2~4)	NPV (sAKI- D2~4)	Specificity (sAKI- D2~4)	AUC-ROC (95% CI)
RAI+	135	0.36 (0.28 0.45)	0.71 (0.59-0.81)			0.89 (0.85-0.94)
RAI-	1,292			0.99 (0.98, 0.99)	0.94 (0.92, 0.95)	
RAI+ and NGAL+	30	0.61 (0.46, 0.75)	0.50 (0.37-0.63)			
RAI- or NGAL-	1,330			0.98 (0.97-0.99)	0.97 (0.97, 0.99)	

Supplemental Table 3 - RAI and NGAL Performance Characteristics to Predict Day 2-4 Severe AKI (sAKI) using data from only patients with a measured and not imputed baseline serum creatinine

Tested result	n	Predicted Values (95%CI)				RAI Performance
		PPV (sAKI+ D2~4)	Sensitivity (sAKI+ D2~4)	NPV (sAKI- D2~4)	Specificity (sAKI- D2~4)	AUC-ROC (95% CI)
RAI+	82	0.37 (0.26-0.48)	0.60 (0.45-0.74)			0.83 (0.76-0.90)
RAI-	595			0.97 (0.95-0.98)	0.92 (0.89-0.94)	
RAI+ and NGAL+	26	0.58 (0.37-0.77)	0.38 (0.23-0.54)			
RAI- or NGAL-	613			0.96 (0.94-0.973)	0.98 (0.97-0.99)	

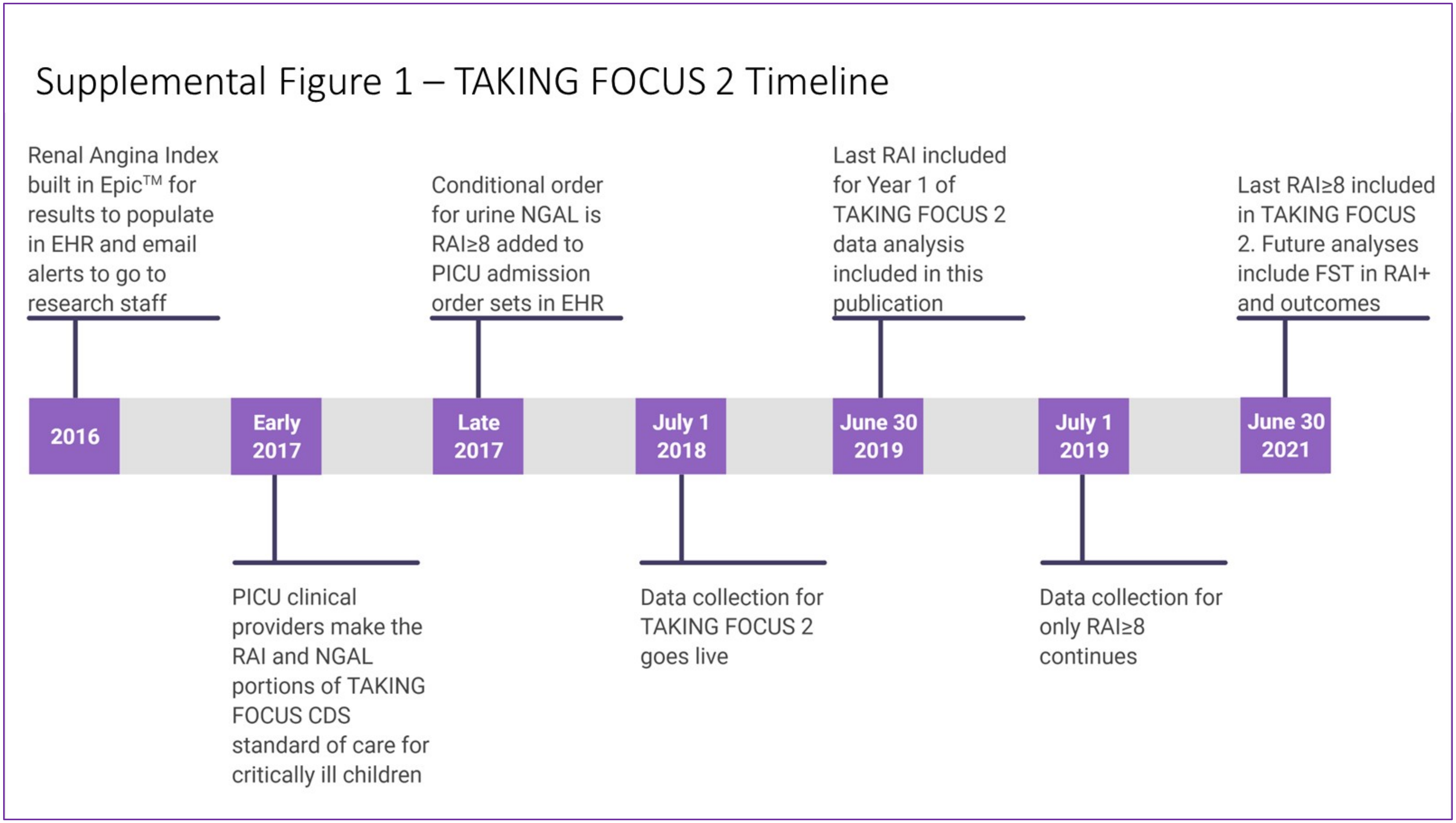

EPIC™ - Electronic Health Record (EHR), Renal Angina Index (RAI), Neutrophil Gelatinase Associated Lipocalin (NGAL), Furosemide Stress Test (FST).
